# A Leak in the Academic Pipeline: Identity and Health Among Postdoctoral Women

**DOI:** 10.3389/fpsyg.2019.01297

**Published:** 2019-06-04

**Authors:** Renate Ysseldyk, Katharine H. Greenaway, Elena Hassinger, Sarah Zutrauen, Jana Lintz, Maya P. Bhatia, Margaret Frye, Else Starkenburg, Vera Tai

**Affiliations:** ^1^Department of Health Sciences, Carleton University, Ottawa, ON, Canada; ^2^Melbourne School of Psychological Sciences, University of Melbourne, Melbourne, VIC, Australia; ^3^Max Planck Institute for Chemical Physics of Solids, Dresden, Germany; ^4^Department of Physics, Technical University Munich, Munich, Germany; ^5^Department of Earth and Atmospheric Sciences, University of Alberta, Edmonton, AB, Canada; ^6^Department of Sociology, University of Michigan, Ann Arbor, MI, United States; ^7^Leibniz-Institut für Astrophysik Potsdam, Potsdam, Germany; ^8^Department of Biology, University of Western Ontario, London, ON, Canada

**Keywords:** academia, women, postdoctoral, social identity, mental health

## Abstract

Several challenges (e.g., sexism, parental leave, the glass ceiling, etc.) disproportionately affect women in academia (and beyond), and thus perpetuate the leaky pipeline metaphor for women who opt-out of an academic career. Although this pattern can be seen at all levels of the academic hierarchy, a critical time for women facing such challenges is during the postdoctoral stage, when personal life transitions and professional ambitions collide. Using a social identity approach, we explore factors affecting the mental health of postdoctoral women, including identity development (e.g., as a mother, a scientist) and lack of control (uncertainty about one’s future personal and professional prospects), which likely contribute to the leak from academia. In this mixed-method research, Study 1 comprised interviews with postdoctoral women in North America (*n* = 13) and Europe (*n* = 8) across a range disciplines (e.g., psychology, physics, political science). Common themes included the negative impact of career uncertainty, gender-based challenges (especially sexism and maternity leave), and work-life balance on mental and physical health. However, interviewees also described attempts to overcome gender inequality and institutional barriers by drawing on support networks. Study 2 comprised an online survey of postdoctoral women (*N* = 146) from a range of countries and academic disciplines to assess the relationships between social identification (e.g., disciplinary, gender, social group), perceived control (i.e., over work and life), and mental health (i.e., depression, anxiety, stress, and life satisfaction). Postdoctoral women showed mild levels of stress and depression, and were only slightly satisfied with life. They also showed only moderate levels of perceived control over one’s life and work. However, hierarchical regression analyses revealed that strongly identifying with one’s discipline was most consistently positively associated with both perceived control and mental health. Collectively, these findings implicate the postdoctoral stage as being stressful and tenuous for women regardless of academic background or nationality. They also highlight the importance of disciplinary identity as a potentially protective factor for mental health that, in turn, may diminish the rate at which postdoctoral women leak from the academic pipeline.

## Introduction

Imposter syndrome. Sexism. Maternity leave. The glass ceiling. The glass cliff. These are just a few of the challenges that disproportionately affect women in academia and perpetuate the leaky pipeline metaphor (Goulden et al., 2011; Resmini, 2016) for women who opt-out of an academic career. Although this pattern can be seen at all levels of the academic hierarchy, the postdoctoral stage seems to be a critical time for women facing such challenges, when personal life changes and professional ambitions often collide. Indeed, this appears to be a time when women are particularly at risk of exiting the academy (Martinez et al., 2007).

In addition to career interruption or relinquishment, the toll taken by this career phase is undoubtedly psychological. Many of the issues facing postdoctoral women involve identity development (e.g., as a mother, a scientist, etc.; Goulden et al., 2011) along with a sense of uncertainty about control over one’s future personal and/or professional prospects (Larson et al., 2014). These challenges, coupled with institutions that lack equitable structures and policies for women (e.g., Lundine et al., 2018), create a climate ripe for career abandonment.

In this mixed-method research, we explore mental health among postdoctoral women, aiming to understand their experiences qualitatively (Study 1) and quantitatively (Study 2). At the heart of this experience, we argue, is a sense of identity. We explore how various social identities—that is, the value and importance of those group memberships to the self-concept (Tajfel and Turner, 1979)—may exacerbate mental health issues among postdoctoral women when they are experienced as not fitting with the academic environment (Iyer et al., 2009). Conversely, we also examine whether identity may protect mental health in ways predicted by the social identity approach to health (Jetten et al., 2012; Haslam et al., 2018).

### Barriers to Workplace Gender Equality

Women in academia face many barriers to workplace equality, which can result in “leaking” from the academic pipeline. The leaky pipeline is a metaphor often used to describe the loss of women in STEM (i.e., science, technology, engineering, and math)—and arguably other fields before reaching senior roles (Goulden et al., 2011; Resmini, 2016; Howes et al., 2018). Although research has yet to determine which barriers contribute most to the leaky pipeline, there are several likely candidates. For example, the well-known metaphor of the glass ceiling—defined as a barrier that results from gender or race and prevents one from moving past a certain point in their career (e.g., promotion or hiring) (Cotter et al., 2001; Bruckmüller et al., 2014)—undoubtedly also affects women in the academy. Further to the glass ceiling, the glass cliff phenomenon (Ryan and Haslam, 2005) may also set women up in precarious leadership roles where they are more likely to fail, and there is reason to believe that this occurs in academia as well, where leadership positions abound (e.g., in teaching, research, and senior administration).

In light of coming up against the glass ceiling or perching precariously on the edge of the academic glass cliff, it should not be surprising that many women in academia suffer from imposter syndrome—the feeling that one is not worthy or competent despite evidence to the contrary. This “syndrome” is more common in women than men and is also associated with attributions that women place on their successes (Clance and Imes, 1978; Howe-Walsh and Turnbull, 2016). Women often attribute their success to temporary causes such as luck, while men more often attribute their success to stable qualities within themselves (Clance and Imes, 1978). Unfortunately, imposter syndrome can start early in a woman’s educational trajectory and may also explain, at least in part, women’s underrepresentation in STEM (and other) disciplines, as well as take a significant psychological toll.

Beyond subjective experiences, there is objective evidence that women are not valued to the same extent as their male counterparts—sexism, gender pay inequity, and fewer chances for promotion also continue to be barriers to workplace equality that women in academia (and beyond) regularly face (Cohen and Huffman, 2007; Savigny, 2014; Lee, 2015; Kohout and Singh, 2018). Women also have less chance of being hired in the first place compared to their male counterparts (Savigny, 2014). For example, when presented with two identical Curriculum Vitaes (CVs) with gender-identifying information included, 127 professors from various fields (physics, chemistry, and biology) considered those belonging to men to be better; however, when the CVs were gender-blind, women were evaluated as better (Moss-Racusin et al., 2012). Indeed, despite the idea that academics or intellectuals are more aware of social injustices and act as critics and the conscience of society (Martin, 1984; Rodden, 2014; Harre et al., 2017), gender inequality occurs in academia, as elsewhere.

Although leaks in the pipeline can be attributed to gender bias and discrimination, another major factor driving the loss of postdoctoral women from academia seems to be whether, and when, they have children (Resmini, 2016; Ledford, 2017). It has been reported that married women with young children are 35% less likely to get a tenure-track position than married men with young children, and are still 33% less likely to achieve this position than are single women without young children (Goulden et al., 2011).

The above-mentioned challenges can be seen at all levels of the academic hierarchy, but a critical time for women facing such barriers is during the postdoctoral stage, when personal life changes and professional ambitions often collide (Martinez et al., 2007). Not surprisingly, such challenges and inequalities—sometimes compounded over time—can take a toll on women’s mental health and perceived control over their career and life trajectories (Kinman, 2001). While these mental health challenges (and gender disparities) are recognized at the graduate student level (Evans et al., 2018), support for postdoctoral fellows often falls through the cracks (Newsome, 2008). It is at this time—the pre-tenure track, postdoctoral stage when women are on the academic job market—that the pipeline appears to be at its leakiest (Martinez et al., 2007; Newsome, 2008; Goulden et al., 2011).

### Challenges for Postdoctoral Women in Academia

One’s time as a postdoctoral fellow—often termed a “postdoc”—can be both challenging and rewarding. However, there are many factors that affect the path of a postdoc’s career, including the balance between these challenges and rewards. On the rewarding side, the hours are often flexible and one can typically pursue a research agenda without the added responsibilities of teaching and administration. On the challenging side, however, those flexible hours are often long, and the promise of a tenure-track or permanent position is uncertain (Larson et al., 2014). Additionally, and regrettably, many postdoctoral women work in environments where they are not respected or supported as women or as academics, particularly in male-dominated fields (Case and Richley, 2013). Many postdoctoral women report experiencing accumulated disadvantages as well as subtle, biased sexism, both of which can combine to create a workplace where women do not feel equal (Steinke, 2013) and don’t perform to their best capabilities (Schmader, 2002). Unfortunately, those perceptions of inequality are often grounded in reality for postdoctoral women. For example, there appears to be a persistent discrepancy in women and men’s publication rates in high profile journals (Lundine et al., 2018)—a marker which, of course, is used among academic employers to measure both the quantity and quality of a potential candidate.

As alluded to earlier, another reason why a woman may have fewer publications than her male counterparts is maternity leave (Goulden et al., 2011; Resmini, 2016). In this regard, timing is critical, given the life-stage that coincides with building the all-important publication record as a prerequisite for securing academic employment. It should not be surprising then that men are considerably more likely to be hired into permanent academic positions compared to women who take maternity leave within 5 years of completing their doctorates (Mason and Goulden, 2004; Resmini, 2016). This overlapping timeline can create an especially steep challenge for women in postdoctoral positions, who have not yet secured permanent employment (Ledford, 2017).

Maternity leave may also have a signaling cost for a postdoctoral woman, suggesting to potential employers that she is not committed to her work (Goulden et al., 2011). Moreover, in many cases the funding supporting postdocs does not allow for paid parental absence, or postdocs are explicitly discouraged from taking leave at all (Ledford, 2017). Maternity leave may also change a woman’s own perceptions and ambitions in career choice: 44% of women reported that issues related to children were key influences in their decision not to pursue a professorship with a research focus (Goulden et al., 2011). Indeed, historically, societal gender norms have dictated that women will give up their careers to have children and devote their time to caring for them. While this is still a laudable path for some women today, those who wish to have children while pursuing an academic career often face a “baby penalty,” which can manifest itself in several ways (e.g., lower quantity of academic outputs, less prestigious or fewer tenure-track job offers, etc.; Mason and Goulden, 2004).

Finally, whether involving children or not, the postdoctoral phase is also a transitional time marked by uncertainty. Uncertainty about one’s future career is often stressful. This uncertainty can be accompanied by a lack of perceived control, which can negatively impact performance (Perry et al., 2005). Moreover, lack of control can negatively impact mental health, by increasing stress and anxiety (Michie, 2002). The lack of perceived control in relation to one’s career prospects, combined with uncertain perceptions about one’s future life in general (e.g., moving cities, starting a family), may encourage postdoctoral women to opt-out of an academic career before it even begins, thereby perpetuating the leaky pipeline metaphor.

Indeed, the barriers and challenges noted here, coupled with institutions that lack equitable structures and policies for women, create a climate ripe for career abandonment during the postdoctoral stage. Beyond practical implications for career progression, however, these challenges also take a significant psychological toll on women at a vulnerable point in their career. We investigate this psychological impact in the present research, focusing on exploring the mental health of postdoctoral women. In doing so, we adopt a theoretical perspective that highlights the importance of identity in guiding and shaping mental health. Not only is a strong sense of connection and identity important to mental health (Jetten et al., 2012), identity is at the heart of many challenges experienced during the postdoctoral stage, when tenuous career conditions may throw women’s social group identities—for example, as a scientist, as an academic, or as a wife or mother—into question.

### A Social Identity Approach to Mitigating the Challenges of Postdoctoral Women

As postdoctoral fellows—a time rife with important life and career transitions—women must often balance multiple social identities. In the context of life transitions, these identities can be both a blessing and a curse. On the one hand, perceived incompatibility between different identities (for example, as mother and employee) can undermine well-being (Iyer et al., 2009). On the other hand, an emerging line of research drawn from the social identity approach (Tajfel and Turner, 1979; Turner et al., 1987) reveals that meaningful social identities can protect mental and physical health. This line of basic research, colloquially termed ‘the social cure’ (Jetten et al., 2012; Haslam et al., 2018), is the foundation of an emerging applied agenda that aims to put this theory into practice to improve mental health outcomes in a variety of populations, including clinical and organizational settings (Haslam et al., 2003, 2016).

Numerous studies have found that group identity can mitigate stressors in an individual’s life, including academic stressors among minority group members (Oyserman et al., 2006), high-impact situational stressors (Haslam and Reicher, 2006), and stressful life transitions (Praharso et al., 2017; Seymour-Smith et al., 2017) such as marriage or becoming a parent. This protective effect is thought to be due to a number of factors, including a robust support network provided by other group members, but also from a sense of collective- and self-esteem drawn from belonging to the group in question (Jetten et al., 2012). Indeed, identifying with a group has also been shown to protect the self-esteem of women exposed to blatant sexism (Spencer-Rodgers et al., 2016), something that women in academia are known to face. This protective effect may even impact individuals at the neurochemical level, in turn, better protecting mental health (Häusser et al., 2012). Likewise, identification with groups can help people feel more in control of their lives, which has been shown to be associated with more positive mental health outcomes (Greenaway et al., 2015). The postdoctoral stage is a time in which individuals might feel that their career prospects—and even their personal life—are out of their control. Identifying with important or supportive groups might help to mitigate such stress and uncertainty, in part due to the social support and sense of community derived therein (Jetten et al., 2012).

What differs in the postdoctoral stage, however, is the uncertainty of those identities themselves (e.g., “Will I ever get a tenure-track job?”, “Will I be able to publish papers while on maternity leave?”). Moreover, some of the identities in question may seem to be incompatible or have conflicting goals (Iyer and Ryan, 2009; Cruwys et al., 2016; Matschke and Fehr, 2017), forcing women into a juggling act where the demands on their time and their resources are not realistic. Such conflicts are sometimes termed identity interference (Settles, 2004). In this regard, female academics may feel that one identity (e.g., scientist, academic) cannot be expressed at the same time as another (e.g., woman, mother; Settles, 2004; Steinke, 2013). Although such identity complexity (Roccas and Brewer, 2002) is not uncommon, it may be especially problematic for women who are simultaneously attempting to establish themselves in an academic career and embarking upon important personal life transitions. In the midst of this conceivable identity incompatibility, women may be less inclined to identify with some group memberships (Matschke and Fehr, 2017) to reduce identity interference, with potential consequences for mental health (Cruwys et al., 2016; Sønderlund et al., 2017).

## The Present Research

The leaky pipeline persists. Women are leaving the academic track at rates greater than men, and this is happening for a variety reasons. In the current research our purpose is not to determine whether women face greater challenges than men in academia—this has already been established. Instead, our goal is to explore nuances regarding the mental health experiences of women during the postdoctoral period—a demanding career stage in which women attempt to secure higher positions in academia. We assess these experiences qualitatively (to capture health experiences in women’s own words) and quantitatively (to examine those health experiences against norms). Moreover, we assess factors that might act as a psychological safeguard against the poor mental health that can stem from the stressors and challenges inherent to this phase of life and career. Specifically, we assess whether group identity (e.g., identifying with other members of one’s discipline or gender) is positively associated with perceptions of control (over work and life) and mental health (e.g., depression, anxiety, stress, life satisfaction).

To examine these research questions, we took a mixed-method approach that combines the empirical rigor of quantitative analysis with the rich contextual insights gleaned from qualitative analysis. Study 1 comprised interviews with postdoctoral women in North America and Europe across a range of disciplines to explore their lived experiences from their perspectives. Study 2 comprised an online study of early career researchers from a range of academic disciplines (e.g., psychology, physics, political science, etc.) and across several countries (e.g., Germany, Australia, United States, etc.) to assess social identification with important groups (i.e., discipline, gender, social), perceived control (i.e., work and life), and mental health (i.e., depression, anxiety, stress, life satisfaction). At the heart of both of these studies is an interest in identity and mental health among postdoctoral women, and how these factors interrelate. In this research we therefore extend and bridge previous work by examining relations among gender inequality, important social identities, and mental health outcomes during a potentially fragile career stage for women from various academic backgrounds and nationalities.

## Study 1

Our aim in Study 1 was to qualitatively explore the challenges faced by postdoctoral women. We interviewed women from a variety of disciplinary backgrounds in both Canada and Germany, to capture their perspectives as well as to gauge differences and similarities across their experiences. Of particular interest were women’s career goals and intentions, gender issues in the academy, women’s experiences of health and well-being (or lack thereof), and potential strategies to alleviate challenges they had encountered.

### Method

#### Participants and Procedure

We conducted semi-structured interviews on two university campuses—in Canada and in Germany, across a variety of disciplines. The Canadian sample consisted of 13 participants ranging from 30 to 44 years of age; 5 of these participants had children, most of whom took less than 1 year of maternity leave (despite the norm in Canada being one full year). These Canadian women had been in postdoctoral positions ranging from 1 to 2.5 years. The European sample consisted of 8 participants, ranging from 29 to 46 years of age; only 1 of these participants had children, and took a 3-month maternity leave (whereas the norm in Germany is also 1 year). Although most of these women had been in a postdoctoral position for approximately 2 years, some had completed multiple postdocs spanning up to 13 years^1^.

We recruited participants through personal emails, institutional postdoctoral contact lists, and recruitment notice postings. The inclusion criteria included being a woman and currently holding a postdoctoral position. After collecting informed consent, we interviewed participants using a semi-structured format that included questions developed from the study’s aims. We used an interview method to allow for a richer understanding of women’s experiences in academia while protecting the anonymity of their responses. We conducted the interviews in Canada between June and August 2017 and each interview lasted between 22.51 and 50.14 min. We conducted the interviews in Germany between August and September 2017, and they lasted between 27.01 and 92.23 min. We conducted all interviews in English, audio-recorded them using Audacity software, and manually transcribed them. Our theory-driven thematic analyses (Boyatzis, 1998; Fereday and Muir-Cochrane, 2006) were facilitated by using NVivo software. We offered each participant a coffee shop gift card as compensation. Names and disciplines have been redacted to avoid identifying participants (as in some cases, the participant was the only female postdoctoral fellow in their department).

### Results

#### Data Analyses

Three independent members of the research team read and coded the interview transcripts. Following this process, we created new themes and we removed or merged redundant themes. We analyzed interviews both as a function of country (Canada vs. Germany) and then together^2^. The result was a finalized list of four main themes, which often intersected with one another, namely (1) career flexibility vs. uncertainty, (2) gender-based challenges, (3) work-life balance and health, and (4) social support and identity (or lack thereof). We consider each of these in turn.

#### Career Flexibility vs. Uncertainty

##### Flexibility and independence

When we asked about their general experiences and what they liked about their current position, postdoctoral women in our sample often noted flexibility, both in terms of their working hours in order to “juggle responsibilities” and in terms of developing new research ideas. As one Canadian postdoc reported: “*What I really like is that I have the flexibility of hours……… I feel that having that autonomy allows me to drive my research forward so I come up with new publication ideas.*” This flexibility was often complimented by a newfound feeling of independence, as another Canadian postdoc noted: “*Once you graduate from the Ph.D. you are no longer a student… you are considered more as a colleague. And I definitely saw that, it was a really welcoming change.*”

However, in line with our theorizing that postdoctoral fellowships can be “the best of times and the worst of times,” several women also explicitly commented on this dichotomy. For example, as one Canadian postdoc reported: “*The flexibility, it’s both a blessing and a curse really, every day you kind of plan for yourself, and it’s a blank slate. But admittedly a lot of times I wake up and I’m not sure what I’m going to achieve that day and I don’t achieve anything*.” Likewise, a similar contrast was noted regarding the independence of the postdoctoral life—although this independence was appreciated in terms of juggling other plans and commitments, there was a downside when considering how postdocs “fit in” to the larger research group from both social and project perspectives. As noted by another Canadian postdoc: “*I have a lot of independence, and that’s really great. I don’t have to call in sick if my little girl is sick, I don’t have to ask to go on vacation. I just do it. No one is questioning me on those things. But on the flipside… being a postdoctoral researcher can be almost too independent at times. Because you may be the only one working on that topic. Depending on the group dynamics, you may be part of the group but you’re not actually integral to anyone else’s research. And so, you may actually just kind of float, which can be good and bad depending on how you look at it. But I think that’s a real risk for most postdocs…*”

##### Uncertainty

Also in line with the postdoctoral experience as “the worst of times,” one of the most prominent themes that emerged from analysis of the interview data was the career uncertainty that postdoctoral women felt. When asked about their career aspirations, even women who indicated that they expected to meet their career goals were still cognizant of the lack of permanent (including tenure-track) positions available within academia. As one Canadian participant noted: “*Well, there are no jobs. You have to move really far to get a job in academia as it is right now. And I’m getting a bit tired of that because I’ve already traveled a lot. So I’m reassessing right now, to be honest, because it’s not clear if there will really be a job in academia in the future. I don’t know if that’s really a realistic aspiration to hold on to*.”

Moreover, many of the women expressed the need to keep their “*options open and not get tunnel vision*,” had already abandoned the idea of pursing a tenure-track professorship because “*there are so few jobs that the reality is most of us are not going to move on in academia*,” had decided to pursue a career in research industry or government, or felt that they were at the midpoint of making that decision. As one German postdoc reported: “*So, when I moved here, at the beginning I really wanted to stay in science…. But now I should be realistic, maybe I don’t have a chance to become a professor especially in Germany – because I want to stay in Germany. And… I’m also thinking about industry. Almost ninety percent I’ve made my decision, and… the problem is, I don’t know about the opportunities in industry.*”

This uncertainty about the future was, in some cases, exacerbated by the uncertainty of women’s present positions, especially in terms of occupying multiple postdocs. This seemed to be a prominent issue for our German interview respondents who had, on average, been in postdoctoral positions for a greater number of years than the Canadian postdocs interviewed. As one German postdoc said: “*So, I have always worked at [this institution]. Since September [year] in something called this “wissenschaftliche Mitarbeiter” [research assistant] position. But my funding has constantly changed... So, my projects have changed regularly but my position is still always the same. I am a temporary – I mean, not temporary, I mean to say, I don’t have a “feste Stelle*” *[permanent position].*

#### Gender-Based Challenges

##### Sexism (and being out-numbered)

In addition to general feelings of uncertainty, some postdoctoral women also reported uncertainty in their own abilities (including “imposter syndrome”), or uncertainty regarding whether some of the attention they received was due to their ability as a scientist or due to their gender, especially in male-dominated disciplines. For example, as a German postdoc reported: “*In certain conferences… it’s not clear whether when you talk to a new person, whether they are flirting with you or they’re not… because there are very few women, sometimes it’s kind of hard to tell the difference between whether they talk to me because they take me seriously as a scientist or because they talk to me because I’m one of the few women in the meeting*.”

Indeed, especially among postdocs in natural science disciplines, many noted that they were often the only woman in the room—an observation that was sometimes missed by their male colleagues, as noted by a Canadian postdoc: “*I’ve been in numerous field camps where I’m the only woman, I’ve been in numerous labs where I’m the only woman, I’ve been in numerous meetings where I’m the only woman… in our particular group there is definitely a male bias. What I find fascinating is that it’s often not recognized by our male colleagues. And I’ve actually been in a room where a male colleague has turned to me and said ‘Do you really think that female directed scholarships are necessary?’ And I kind of looked at him like ‘Are you effing kidding me? And I looked around… and I was the only female in the room*.” In some cases, this lack of recognition of gender disparity by male colleagues specifically highlighted the leaky pipeline at the postdoctoral stage, as noted by another Canadian postdoc: *“I’ve had male counterparts… [say] ‘Well how is there a gender bias? I can’t even find any male students, all my grad students are female.’ It’s like ‘Yeah, but they’re at the Master’s level, how many female postdocs do you have? Oh none?”*

Interestingly, alongside the challenges of not being taken seriously and networking with male colleagues without being misinterpreted as having romantic interests, many of the women we interviewed commented that “*you just get used to*” being in male-dominated environments. Unfortunately, however, other instances of gender-based challenges constituted more overt sexism, often based on the assumptions that women were weaker, both physical and mentally, did not belong in the discipline, or were not prepared for an academic career. A telling example came from one Canadian postdoc when discussing support from her supervisor regarding her job prospects: *“I think the assumption is just that I’m not going to academia, and so I’ve had a few [times] where [my] male counter parts are… involved in more projects, and more taken under the wing, and so they end up being on more papers. And it’s just assumed that they’re heading into academia. And for me, they are… nice and supportive, but when it actually comes to inclusion in projects and planning and papers and writing, I get forwarded jobs that are not academic; I get forwarded jobs that are more [entry] level; I get forwarded jobs that are not academic stream. I’ve always been fascinated by that, because we’ve never had a conversation about my choices.”*

Likewise, some female postdocs noted being “put down” or “looked down upon”. Others noted being subject to inappropriate comments, as noted by a German postdoc, “*in some environments people will never make any inappropriate comments, but… some people in other environments will, you know, say… ask whether she’s good looking or… just talk about it as if it’s a normal topic to discuss, when you’re talking about a scientist*.” In many cases, sexist attitudes also intersected with race and age, especially in male-dominated disciplines. A Canadian postdoc noted: “*I went through working in a very male dominated career... a lot of the people that are in charge in that realm are older white men. So there’s a specific culture that’s created… there’s a real intersection between the age and gender thing. And there are these… hierarchical judgements*.”

Finally, several of the postdocs interviewed commented on the gender disparity between men and women in terms of their geographical mobility. Specifically, they noted that, in their experience, male colleagues had more freedom to travel—both short-term and long-term—than did women. A German postdoc explained: “*It’s more common that… men will be in a relationship and… their partner will… either not have very specific career goals so they won’t mind so much moving, where they are moving, and moving quite often. And well, this is a lot less likely to happen if you are a woman*.”

##### Maternity leave (pregnancy and child-rearing)

Of all the gender-based challenges noted, the challenge of taking maternity leave was most prominent in our analyses of the interview data. Narratives were volunteered from women who had postponed having children, given up various academic pursuits to have children, or felt they had been penalized for taking maternity leave. Indeed, several women noted the feeling of “lagging behind” their peers while on maternity leave, as described by a Canadian postdoc: “*It’s difficult to stay engaged during that year. It’s difficult, and so women are starting to take shorter and shorter mat leaves because they know that they are going to start getting looked over…*”. Likewise, the challenge of nursing an infant contributed to the feeling of falling behind one’s peers in terms of the time needed to remain productive, as noted by another Canadian postdoc: “*I think just the fact that I was breastfeeding… that did create challenges, because it’s harder to be away from the baby and then when I came back to work I’d have to take time to pump milk and there wasn’t really a suitable spot to do that. So it would take up a big part of my day.*” These concerns emerged in light of perceptions that the academic job market does not take the loss of productivity during a woman’s maternity leave into consideration. As explicitly noted by a Canadian postdoc: “*There’s [a] challenge if you decide to have children. I feel like that does harm your career. Because I don’t think it’s recognized… you’re still expected to be producing a certain number of publications even if you are taking time off to have kids..*.” Although some potential solutions were offered, ranging from offering more paternity leave to freezing women’s eggs, concerns were expressed that “*I still don’t know how [hiring committees] could change their criteria to make it more fair.”*

While only 6 of the 21 women that we interviewed for this project had children, many of those who did not have children also stated that one of the biggest challenges for a female postdoc is choosing between having children (and when) and progressing in their career. This sentiment was poignantly expressed by one postdoc, who stated, “*Particularly since I haven’t had any kids, I’m still safe.*” Indeed, several women we interviewed stated that they would like to have children, but felt the need to factor their work into the decision of when to have them. This conflict was described by a Canadian postdoc, saying: “*You sort of reach the postdoc stage where you’re supposed to launch academically at the same time that you have to decide whether or not to have kids. And most women have put it off because they’ve been studying so long… I mean, I’m facing that decision myself now, it’s now or never*.”

Likewise, women noted perceptions of stigma associated with taking leave itself, including the notion that having children was “frowned upon” or discouraged. As a German postdoc reported: “*The gossip in my department was that… the climate was not very conducive for women to become pregnant and take time off. I mean… so that then they become less useful for the department during their time off… if you do become pregnant… you’re falling out of favor with your boss…. Even though my boss didn’t say anything to me personally, but just that his behavior to other women prior to me, who had experienced that in the group… showed me that… the outcome might not be the best*.” Moreover, some of the challenges of having children intersected with blatant discrimination on this basis, as noted by a Canadian postdoc: “*There’s still the perception like that it’s viewed negatively if you decide to have kids. Like I was told for a position I was looking into, ‘I don’t think that you’d actually be able to do the project because of your family situation’… which I’m pretty sure [is] one of the things you’re not really supposed to say in an interview situation*.”

Importantly, the challenges of maternity leave were not restricted to the leave itself, as women also reported that the challenges of pregnancy (e.g., fatigue, etc.) potentially hindered postdocs’ productivity. However, this was especially evident among women in the natural sciences, whose lab work often had to cease as soon as they became pregnant, as noted by a German postdoc: “*No matter how nice, no matter how helpful the father is, it’s always easier for the guys to have kids than for the girls. Because for girls – especially experimentalists – we can’t be in the lab for like a year. So, in a year, you literally cannot work on your samples so then, yeah you could write, you can do some other things, but you’re [going to] be set back…. Cause the first year… even while you’re pregnant, your life is completely messed up and the guy would not be affected*.”

And finally, on the other side of maternity leave, women noted that child-rearing and the “emotional labor” of mothering was a continuing challenge—not only compared to men (as many also acknowledged their husbands’ help in this regard), but also in comparison to other women without children. As explained by a German postdoc: “*Making [your] career is really a challenge because it is the woman who has to have the children. Conceive, carry and deliver and, the first months… when children are 1 year old, 2 years old, they need also still a lot of support. And by nature, or by individual preference, women are doing a lot of stuff for the children. I don’t know why – in principle, men can also do that, but I think women voluntarily do it. Because… of nursing and all these things they are more connected to the children. So, if you have a demanding job… in my own experience… it is extremely difficult to compete with people who have no children or men*.”

#### Work-Life Balance and Health

##### Work-life balance

Intersecting with gender-based challenges—including having children—the postdoctoral women in our study reported the challenge of achieving so-called work-life balance, especially in light of the uncertainty of the competitive academic job market. However, unlike the aforementioned flexibility of work hours and idea development, they discussed a stark contrast in the flexibility of academia itself, especially as it related to balancing other aspects of life and family. As reported by a Canadian postdoc: “*There’s no flexibility in academia, there’s no acknowledgment of the importance of family life, or anything like that. You are either expected to play the game in full or get out*.” Nonetheless, some of the postdoctoral women we interviewed were attempting to achieve this balance, as described by another Canadian postdoc: “*I definitely have faced challenges in terms of work-life balance. My husband’s not an academic, and he thinks I work a ton. And he has tried to lay out ground rules of when I can and cannot work, which… is a healthy thing. But that’s a challenge… balancing that.*”

Interestingly, several of the German postdocs—especially those who had lived in multiple countries—noted a discrepancy in work-life balance between Europe and North America, with many opting to stay in Germany (or Europe more broadly) in hopes of maintaining more balance. As one German postdoc put it: “*That’s the… difference… between the US and Germany or Europe: there’s a better life-work balance in Europe as opposed to the US…. in the US, majority of people are workaholics. And that kind of inspires this culture of ‘if you don’t stay in the lab for 12 h a day you’re a horrible person,’ which is not always true because there is only… so much time that you could be efficient.*”

The women interviewed in Canada and Germany alike, however, expressed frustration with the timing of the postdoctoral stage in terms of achieving work-life balance or planning one’s personal life. As stated by a German postdoc: “*I don’t think science is very ideal in the whole lay-out, in how you’re supposed to proceed with your career. At age thirty, should I really have a temporary job where I’m working in a city for 2 years and then I’m expected to move to a whole new country, again? This is crazy. I can’t – and then I’ll be 34 years old on my second post-doc in some temporary city – how are you supposed to have a family like this? I’m not doing a second post-doc, there’s no chance in hell…”.*

##### Mental and physical health

As anticipated, the postdoctoral women in our study also reported that the lack of work-life balance impacted their mental and physical health. The mental health issues they identified primarily included depression, anxiety, and stress. Moreover, concerns about work-life balance also intersected with identity issues, in that focusing solely on the needs of one’s work identity came at the expense of positive mental health. As noted by a Canadian postdoc: “*If you spend all your time doing something then that becomes sort of part of your identity. So then if things aren’t going well in that aspect then it’s harder to be positive about other things in your life if you associate your main identity [as] being a scientist. Then if you encounter barriers to employment in being a scientist then I think that is a bit of a challenge*.” In some cases, women also explicitly attributed mental health issues to imposter syndrome, as in the case of a Canadian postdoc who reported: “*Mentally I think definitely there’s been some bouts of depression. You know, definitely some imposter syndrome… So with that, you know, definitely some anxiety…*”.

To a great extent, the mental health issues of anxiety and stress also stemmed from the frustration and uncertainty that women felt about their career prospects, noting that “*looking around at my colleagues, everyone has issues… they start developing in the PhD and they just get worse. It’s mostly due to the uncertainty*.” Others recognized that the daily stress they experienced was nearly inevitable in pursing an academic career, and struggled both with achieving work-life balance in their own lives, but also with not promoting that culture to get ahead. As one Canadian postdoc said: “*I think that’s part of the training, but it’s also part of the lifestyle, being an academic, which is terrible, it shouldn’t be, we shouldn’t expect that from people. But it’s strange how the culture kind of… gets in to your own head. You think you know work-life balance is important, but then… I will still secretly judge if somebody always goes home at 4pm, and I know I shouldn’t because it’s great for people to set up their lives however [they] want to. But there is this… highly competitive spirit that everybody sort of expects, that if you want to be the best then you have to work 80 h a week. And that’s just not feasible for most people, and most people don’t want that, but I think that that’s still the number one thing… is this element of stress*.”

Women reported several physical ailments, also often associated with stress, including high blood pressure, exhaustion, stomach issues, back pain, and especially insomnia. Interestingly, even those women who said that they did not experience negative effects on their health due to their academic careers mentioned that they experienced great amounts of stress and contended with sleepless nights, suggesting that those women came to expect extreme stress and lack of sleep as a part of the normal postdoctoral experience. As commented by a Canadian postdoc: *“… you end up working ridiculous hours… and it’s probably not good, and I was probably more stressed than I should have been, or getting less sleep or less restful sleep that I should have been … but nothing outside the norm.*” Likewise, another postdoc downplayed the severity of her mental and physical health symptoms, saying that she experienced “*a lot of stress, due to the lack of a proper schedule. This could be my own fault, but I’m only realizing it now. Insomnia, stomach issues. Just basically anxiety related things*.”

#### Support and Identity (or Lack Thereof)

##### Institutional barriers

The mental and physical health issues experienced by postdoctoral women also intimated at the lack of support available, which was the final theme that emerged from our analyses of the interview data. When we asked about what could be done to help alleviate some of the challenges they faced, greater support from postdocs’ universities or institutions, for mental health issues in particular, was requested. As one Canadian postdoc recounted: “*My officemate actually was particularly anxious and he called some kind of help line at [the university] looking for support and they denied him anything as a postdoc. They told him if he were a student okay, or faculty okay, but as a postdoc we can’t help you….*”

Women also reported other institutional barriers related to health, broadly speaking, as well as those specifically affecting women, especially the Canadian postdocs. In this regard, one woman noted inconsistencies across health coverage, saying that “*we have three different postdocs in our lab, we get paid through three different mechanisms, and we all have three different health insurance coverage because of that.*” However, several of the women we interviewed also noted policies around maternity leave and childcare as an institutionalized gender-based challenge needing to be addressed, in terms of *“… thinking critically about scholarships and how scholarships actually provide maternity leaves….*” A Canadian postdoc expressed her frustration with the system, saying “*I believe the current status is they offer you 4 months of maternity leave, but my understanding is that even… the university here doesn’t take newborns until 6 months. So you have this daycare system that doesn’t take babies until 6 months, and yet you have [scholarships] that only provide maternity leave for 4 months. How does that work?*”

As recounted in our findings about gender-based challenges above, many postdoctoral women also searched for answers regarding how to eliminate gender biases and achieve greater equity in hiring practices. At the same time, many of the women we interviewed—in both Canada and Germany—expressed discontent that although the academy often publicly acknowledges these biases and barriers, they saw few changes in practice. As noted by one of our participants: “*I think it’s fascinating that universities strive… to make statements about… getting their gender bias basically under control and yet when you submit CVs it’s still… this standard form with ‘How many papers have you published?’ ‘What’s the impact factor?’... So structural barriers… are documented and real, and yet the universities still have this gender bias problem*.” Indeed, there appeared to be consensus among the women we interviewed that both institutional as well as broad societal changes were needed to enact change in this regard. In some cases this even included women’s own understanding of the issues, as expressed by a German postdoc who confessed, “*I’m not a feminist… my opinions are very—are on the traditional side sometimes, even though I know that’s bullshit. And then I catch myself and then I have to think and evaluate why I thought that way.*”

Importantly, the postdoctoral women in our study also conveyed that the barriers women face are cumulative, and that the “*tipping point*” is at the postdoctoral level “*where then you have a higher representation of men in academic positions, and… that kind of goes up the chain.*” In particular, the barriers women faced exacerbated the issues of achieving work-life balance or challenges of having children at the postdoctoral stage. As noted by a Canadian postdoc: “*At the postdoc level, where you’ve been in the system for 10 years that has been imposing institutional barriers on you for 10 years—where are you in that? I think for women specifically… I didn’t feel any barriers when I was [in] a Bachelor’s [degree] cause I didn’t want kids then, right? As you move through, it’s at that Master’s, Ph.D., postdoc level that people are having to make choices between having kids or not having kids. And so again they are accumulating over time*.”

##### Support, mentorship, and identity

In the midst of numerous barriers and challenges, the postdoctoral women we interviewed also acknowledged the support they received from friends and family, colleagues, and the importance of having a mentor to guide them. For example, a Canadian postdoc recalled her husband’s support after they’d had a baby, which was augmented by his ability to take paternity leave: “*The only way I was able to advance the way that I wanted to was because my husband was able to take paternity leave at the same time. Because otherwise it would have been really challenging because I didn’t have any paid maternity leave. So him having leave at work was actually really important for me to be able to still publish papers and to be looking for postdocs and that kind of thing*.”

Within the academy, however, many postdoctoral women in our study attributed much of their support to strong female mentors. As recounted by a German postdoc: “*The fact that I had female advisors up until coming here, I think it helped quite a bit. Because my undergrad advisor, she was very young when she started so I basically saw her build her lab up. And then my Ph.D. advisor, she also was very young… and she had two kids while I was in grad school. So, I got to see all the spectrum of… getting a position, building up your lab, going up for tenure, having kids… So it was… very nice to see that she was able to do all that and still do science*.” Likewise, the postdoctoral women in our study reported that being around women who had experienced—and persevered through—many of the same challenges that they currently faced inspired them. As stated by a Canadian postdoc: “*I’ve had a lot of strong women role models around me… Women who, they’ve gone through real challenges of establishing themselves as… the one woman or… having to contend with being dismissed…. And so I’m indebted to the women who came before me who have tackled that and paved [the] way a little bit for me*.”

Nonetheless, women also expressed concerns that the often male-dominated, competitive environment might induce women to forget about the struggles faced by their female predecessors, and that they might lack empathy for the women that come after them. As conveyed by a Canadian postdoc: “*I worry about making sure I avoid doing this myself… like the tough love kind of thing… ‘if I could do it, you can do it,’ but without the kind of compassion and empathy for that…. And then… there’s less of a sense of unity among women*….”

Along with the mentorship of female role models, several of our study participants also commented on the importance of having support mechanisms in place among their colleagues to help during difficult times, including feelings of loneliness and social isolation. As noted by a Canadian postdoc: “*At the postdoc level… I’m reminded how isolating it is.*” Support from other postdoctoral women, as well as those with the same disciplinary background (or both), were reported by women as being vital to achieving a “*feeling of togetherness*,” as noted by one of the German postdocs in our study. This was deemed to be especially important at the postdoctoral stage: “*In academia… I think, especially in this post-doc stage where people move around a lot… your first circle of social activities is with your colleagues also. So this is quite important – that you can get along well with them*.”

Nevertheless, women also expressed concerns that the patriarchal culture in which the academy is embedded impeded women’s support for one another, including the notion that competition to get ahead superseded any sense of “togetherness” that might be brought about by shared gender identity. As a German postdoc suggested: “*We don’t support each other. I think we need… a strong network between women – we should support each other. And even one of my male colleagues said in this workshop, ‘We go outside and drink beer, but you ladies, do you do [things] like this?’ And we were thinking, ‘No… we don’t’. I think even between ourselves we have competition and we are not so much supportive*.”

### Discussion

The findings of this qualitative study suggest that at least some of the challenges encountered by postdoctoral women are similar to those faced by women at other levels of the academic hierarchy (and in other fields of work), including sexism (Savigny, 2014), institutional barriers (Case and Richley, 2013), and the impacts of limited work-life balance on health (Emslie and Hunt, 2009). However, postdoctoral women also faced some unique challenges, which appeared to be driven largely by the uncertainty of both their career and life stages. As noted previously—for women who are attempting to simultaneously transition both personally and professionally—timing matters (Goulden et al., 2011; Resmini, 2016). Although some differences emerged across the two countries (e.g., greater perceptions of work-life balance in Europe compared to North America), and some challenges (e.g., sexism) stood out as more problematic in male-dominated disciplines, the experiences and perspectives of postdoctoral women in both Germany and Canada largely mirrored each other.

In particular, although the postdoctoral women in our study appreciated the flexibility of their current positions, the thread of uncertainty seemed to weave its way through other aspects of women’s lives, including family planning, experiences of depression, stress, and anxiety, as well as other stress-related physical symptoms. Moreover, although the postdoctoral women in our study acknowledged the support of family, colleagues, and female mentors, the competitive nature of the job market also threatened to erode a positive sense of gender identity from which women could draw (and give) support (Haslam et al., 2005). Nonetheless, in the midst of numerous stressors and challenges faced, drawing on such support seemed to be a singular positive aspect of their experiences, suggesting that the support derived from various group memberships may be a fruitful avenue to explore as a potential mitigating factor against postdoctoral women’s mental health distress and disrupted career goals.

## Study 2

Study 1 revealed a number of barriers faced by postdoctoral women that centered on themes of uncertainty, gender-based challenges, work-life balance, and (lack of) support during a critical and stressful life stage. In Study 2, we considered the positive role of social support (e.g., from family, co-workers, role models) in thinking about how these challenges might be mitigated psychologically. The social identity approach has been employed by psychologists in recent years to understand and provide solutions to a range of health and well-being challenges (i.e., “the social cure”; Jetten et al., 2012). A main finding of this literature is that a range of important social identities—the groups to which people belong and with which they identify—are often uniquely beneficial for mental and physical health (Jetten et al., 2012, 2017; Haslam et al., 2018).

Applying this logic to the postdoctoral stage, using a large-scale multi-country survey, we assessed which—and to what degree—various social identities are associated with better well-being among postdoctoral women. In line with social identity theorizing, we predicted that identification with a variety of meaningful groups would be associated with better well-being among postdocs. However, we expected that these associations might differ depending on the identities in question. As revealed in the qualitative analysis, postdoctoral women face conflict between a number of identities, including gender-based, work-based, and, in some cases, parenthood-based identities, each of which might impact mental health to varying degrees. Following from this, in Study 2 we assessed discipline (e.g., physics, psychology, etc.) identification, gender identification, and social group (i.e., friendship) identification as potential protective factors against female postdocs’ mental health issues in the form of lack of work and life control, greater depression, stress and anxiety, and lower life satisfaction.

### Method

#### Participants and Procedure

Participants were recruited using convenience samples from the authors’ social and professional networks, and wider distribution via online mailing lists of several early career researcher networks. Participants completed an online survey using Qualtrics software. The final sample comprised 304 researchers (*M*_age_ = 35.59, *SD* = 6.47). The majority were female (74%) and currently held a postdoctoral position (70%; as opposed to a permanent or graduate student position). Just under half of the sample had at least one child (44%).

Given our focus on the experiences of postdoctoral women specifically, we isolated our analysis to the 146 women in the sample who were currently in a postdoctoral position (*M*_age_ = 33.40, *SD* = 4.45). According to G^∗^Power, this sample size provided us with 92% power to detect medium effect sizes (two-tailed; Faul et al., 2007; Erdfelder et al., 2009). Of this sample, roughly a third (37%) had at least one child. The majority of this sample worked in the natural sciences (80%, e.g., Earth Sciences, Astrophysics, Biology, Medicine), followed by the social sciences (14%, e.g., Psychology, Urban Studies), and humanities/other (6%, e.g., Law, Public Health, Epidemiology). Participants were based in Germany (43%), the United Kingdom (18%), Australia (10%), the United States (6%), Canada (6%), Portugal (4%), France (4%), the Netherlands (2%), Sweden (2%), or other (5%).

#### Measures

##### Depression

We measured depression using seven items adapted from the depression subscale of the Depression, Anxiety, and Stress Scale, short form (DASS; e.g., “I couldn’t seem to experience any positive feeling at all”; Henry and Crawford, 2005). Participants reported the degree to which the items had applied to them during the previous week on a scale ranging from 0, *did not apply to me at all* to 3, *applied to me very much or most of the time*. As is the norm for this scale, we summed the items and multiplied by 2 to form an index of depression.

##### Anxiety

We measured anxiety using seven items adapted from the DASS short form (e.g., “I felt I was close to panic”; Henry and Crawford, 2005). Participants reported the degree to which the items had applied to them during the previous week on a scale ranging from 0, *did not apply to me at all* to 3, *applied to me very much or most of the time*. As with depression, we summed the items and multiplied by 2 to form an index of anxiety.

##### Stress

We measured stress using seven items adapted from the DASS short form (e.g., “I found it difficult to relax”; Henry and Crawford, 2005). Participants reported the degree to which the items had applied to them during the previous week on a scale ranging from 0, *did not apply to me at all* to 3, *applied to me very much or most of the time*. Once again, we summed the items and multiplied by 2 to form an index of stress.

##### Life satisfaction

We assessed life satisfaction using Diener et al. (1985) Satisfaction with Life Scale. The scale comprises five items (“In most ways my life is close to my ideal”), scored on a scale ranging from 1, *strongly disagree* to 7, *strongly agree*. As is the norm for this scale, we summed the items to form an index of life satisfaction.

##### Control over life

We assessed perceived life control using three items (e.g., “I feel in control of my life”; Greenaway et al., 2014) scored on a scale ranging from 1, *strongly disagree* to 7, *strongly agree*. As is the norm for this scale, we averaged the items to form an index of perceived life control, α = 0.84.

##### Control over work

We assessed perceived work control using a scale by Ruthig et al. (2009). The scale comprises eight items (four positively worded, e.g., “The more effort I put into my work, the better I do” and four negatively worded, e.g., “No matter what I do, I can’t seem to do well in my work”) scored from 0, *not at all* to 4, *extremely*. We reverse scored the negatively worded items and, as is the norm for this scale, we summed all items to form an index of perceived work control.

##### Group identification

We assessed participants’ identification with 10 different groups (as well as the option to include another group not listed) using one-item adapted from Postmes et al. (2013; e.g., “I identify as a member of my academic discipline”) scored on a scale ranging from 1, *strongly disagree* to 7, *strongly agree*. We also asked participants to indicate which of the 10 listed groups (or to suggest another) they identified with most strongly.

##### Demographics

Participants provided demographic information including age, gender, whether they had children (coded for analyses as 0 = *no* and 1 = *yes*) and discipline (coded for analyses as 0 = *social science/humanities/other* and 1 = *natural science*).

### Results

#### Preliminary Analyses

Means and standard deviations for the mental health variables are presented in Table 1. These show that female postdocs experienced mild levels of depression and stress, although normal levels of anxiety. Life satisfaction was in the slightly satisfied range, and the control (over work and life) variables had no available normed data, but responses sat on average around the mid-point of the scale, indicating only moderate levels of perceived life and work control.

**Table 1:** Descriptive statistics of mental health variables in Study 2.

	*M*	*SD*	Range of scores	Top of scale	Level
Depression	9.82	8.45	0–40	42	Mild
Anxiety	5.97	6.43	0–32	42	Normal
Stress	13.04	7.47	0–36	42	Mild
Life satisfaction	23.33	6.80	5–35	35	Slightly satisfied
Life control	4.63	1.38	1–7	7	Non-normed
Work control	17.89	4.53	7–27	32	Non-normed

*Levels were determined based on scale norms, where available.*

To select the most relevant group identification variables, we assessed responses to the item that asked participants to indicate which group they identified with most strongly. As seen in Table 2, participants identified most strongly with their social (i.e., friendship) group, work (i.e., disciplinary) group, parental role, and gender group. Because only 37% of participants had children, we omitted parental role from the analyses reported below, and focused on the other three identities as predictors of mental health outcomes.

**Table 2:** Percentages of group importance in Study 2.

	Percent selected as most important group
Social group	31.5%
Work group	28.0%
Parental role	17.5%
Gender group	5.6%
Sports group	4.2%
National group	1.4%
Ethnic group	1.4%
Religious group	1.4%
School group	1.4%
Political group	1.4%
Other group	6.3%

#### Predictors of Mental Health

Bivariate correlations among all variables are displayed in Table 3. These correlations suggested that neither age, having children, nor discipline, were associated with any of the group identification or mental health outcomes. Interestingly, modest positive correlations emerged between gender identity and both social group (*r* = 0.18) and discipline identity (*r* = 0.19, *p*s < 0.05), whereas the latter two identities were unrelated to each other, perhaps suggesting a low degree of identity overlap (and potentially high degree of identity conflict). However, group identification (both with one’s discipline and social group) appeared to play a role in fostering well-being.

**Table 3:** Bivariate correlations among variables in Study 2.

	1	2	3	4	5	6	7	8	9	10	11	12
(1) Age		0.46^∗∗∗^	0.11	0.04	0.04	−0.02	−0.04	−0.03	−0.01	0.02	−0.13	−0.01
(2) Children			−0.09	−0.04	−0.01	0.02	−0.05	−0.13	0.03	0.12	−0.08	0.06
(3) Natural science				0.15	−0.03	−0.08	0.05	0.02	−0.02	−0.08	−0.01	−0.02
(4) Discipline ID					0.19^∗^	0.15	−0.28^∗∗∗^	−0.11	−0.16	0.32^∗∗∗^	0.24^∗∗^	0.32^∗∗∗^
(5) Gender ID						0.18^∗^	−0.05	0.05	−0.17	0.11	0.08	0.11
(6) Social group ID							−.13	0.01	0.01	0.17^∗^	0.23^∗∗^	0.14
(7) Depression								0.49^∗∗∗^	0.62^∗∗∗^	−0.62^∗∗∗^	−0.49^∗∗∗^	−0.43^∗∗∗^
(8) Anxiety									0.68^∗∗∗^	−0.22^∗∗^	−0.23^∗∗^	−0.21^∗^
(9) Stress										−0.39^∗∗∗^	−0.35^∗∗∗^	−0.23^∗∗^
(10) Life satisfaction											0.67^∗∗∗^	0.40^∗∗∗^
(11) Life control												0.41^∗∗∗^
(12) Work control												

*Children coded as 0 = no and 1 = yes. Natural science coded as 0 = social science/humanities/other and 1 = natural science. ID, identification. ^∗^p < 0.05; ^∗∗^p < 0.01; ^∗∗∗^p < 0.001.*

We subsequently conducted a series of multiple regression analyses, regressing the dependent variables (mental health and control) onto age, parenthood, discipline (i.e., natural science vs. not), and the three identification variables of interest: discipline identification, gender identification, and social group identification, in order to further delineate these relations when all variables were accounted for.

##### Depression

Together, the variables accounted for a significant amount of variance in depression, *R*^2^ = 0.10, *F*(6,129) = 2.25, *p* = 0.042. Of the individual predictors, however, only discipline identification was significant, β = −0.26 (95% *CI* = −0.41, −0.10), *SE* = 0.08, *p* = 0.001 (all other βs < 0.08, *p*s > 0.400), such that greater identification with one’s discipline was associated with lower depression.

##### Anxiety

Together, the variables did not account for a significant amount of variance in anxiety, *R*^2^ = 0.03, *F*(6,127) = 0.67, *p* = 0.672, nor were any of the individual predictors significant (all βs < 0.20, *p*s > 0.341).

##### Stress

Together, the variables did not account for a significant amount of variance in stress, *R*^2^ = 0.06, *F*(6,124) = 1.24, *p* = 0.291. However, discipline identification was marginally significant, β = −0.15 (95% *CI* = −0.31, 0.01), *SE* = 0.08, *p* = 0.076 (all other βs < 0.07, *p*s > 0.101), such that greater identification with one’s discipline was associated with lower stress.

##### Life satisfaction

Together, the variables accounted for a significant amount of variance in life satisfaction, *R*^2^ = 0.17, *F*(6,131) = 4.32, *p* < 0.001. However, only discipline identification was significant, β = 0.31 (95% *CI* = 0.16, 0.46), *SE* = 0.07, *p* < 0.001 (all other βs < 0.12, *p*s > 0.211), such that greater identification with one’s discipline was associated with greater life satisfaction.

##### Life control

Together, the variables accounted for a significant amount of variance in life control, *R*^2^ = 0.12, *F*(6,131) = 2.85, *p* = 0.012. Both discipline identification, β = 0.20 (95% *CI* = 0.05, 0.35), *SE* = 0.08, *p* = 0.010, and social group identification were significant, β = 0.16 (95% *CI* = 0.02, 0.30), *SE* = 0.07, *p* = 0.030 (all other βs < 0.07, *p*s > 0.301), such that greater identification with one’s discipline and social group was associated with greater perceived life control.

##### Work control

Finally, the variables together accounted for a significant amount of variance in work control, *R*^2^ = 0.14, *F*(6,128) = 3.36, *p* = 0.004. Once again, only discipline identification was significant, β = 0.31 (95% *CI* = 0.15, 0.47), *SE* = 0.08, *p* < 0.001 (all other βs < −0.24, *p*s > 0.226), such that greater identification with one’s discipline was associated with greater perceived work control.

### Discussion

Alongside identifying a number of barriers faced by women in the critical postdoctoral academic stage through a rich qualitative analysis in Study 1, we aimed to explore these experiences—including their links to mental health—quantitatively in Study 2. We collected an international sample of female postdocs from Europe, Australia, and North America, spanning a number of disciplines but featuring particularly women in the natural sciences who often face additional challenges related to lower representation than their male colleagues. These analyses confirmed the trends described in the qualitative analysis: female postdocs showed somewhat low levels of mental health and only moderate levels of perceived control over one’s life and work. Given the importance of these factors in living a happy and healthy life (Helliwell et al., 2013; Greenaway et al., 2015) women in the postdoctoral period appear at risk for short-term (and potentially long-term) mental health issues.

However, on a positive note, and in line with literature in the social cure tradition (Jetten et al., 2012), we found that group identification was a protective factor, yielding better mental health on almost all surveyed outcomes. Critically, only identification with one’s discipline (i.e., as a biologist, as an epidemiologist, and so on) uniquely served this protective function consistently. Although women reported being relatively highly identified with their social and gender groups, these identities did not predict unique variance in mental health outcomes with the exception that greater identification with one’s social group was associated with greater perceived life control. In this regard, it may be that identification with a closely related domain (discipline vs. social group) offered greater perceptions of control over that area (i.e., work vs. life control, respectively). However, these findings also corroborate other observations in earlier academic spheres—for example, among undergraduate students—that discipline (but not gender) identification is associated with positive outcomes, such as deeper approaches to learning (Smyth et al., 2015) and working memory (Rydell et al., 2009). These insights suggest that, at least in this case, fostering a sense of identification with one’s discipline or area of study may protect the mental health of women in the challenging postdoctoral stage.

## General Discussion

Our findings from the two studies reported here suggest that postdoctoral women face an array of unique challenges that may precipitate their leak from the academic pipeline. These challenges are both practical and psychological in nature. More positively, our findings also suggest that identifying strongly with others—especially members of one’s disciplinary group (i.e., as a physicist, political scientist, biologist, etc.)—is associated with better mental health during this tumultuous career period. We were particularly interested in the mental health experiences of postdoctoral women who were currently navigating the academic job market. As expected, these women reported a high degree of career uncertainty and numerous gender-based challenges, both of which exacerbated lack of work-life balance and mental health distress. Although the redeeming quality of their experiences stemmed from the social support and sense of identity they shared with others, even this appeared to be tenuous within the competitive nature of the academic environment. Given that these factors have not (to our knowledge) been examined in concert, this research makes a novel contribution to literature on the “social cure” (Jetten et al., 2012) by examining these issues among postdoctoral women—who are at a critical time in both personal life and career development. More broadly, the findings suggest several avenues through which gender inequality within the academy might be reduced.

### Implications for Postdoctoral Women’s Health and Well-Being

Although we expected that group identification would be associated with better well-being among female postdocs, we also predicted that these associations might differ depending on the identities in question. Indeed, as seen in Study 2, only disciplinary identification consistently predicted positive mental health (in the form of fewer symptoms of stress and depression, as well as greater life satisfaction and perceived control). This finding was striking, especially in light of the many challenges reported by postdoctoral women within male-dominated fields in Study 1, and given that a large part of our online sample also comprised women in (often male-dominated) natural science disciplines (Young et al., 2013). Importantly, however, the type of discipline (i.e., natural science, social science, humanities) was unrelated to the mental health indicators assessed; instead, strongly identifying with one’s discipline—no matter what area of study—appeared to offer benefits.

These findings hold promise for the mental health of postdoctoral women who are able to feel a sense of belonging and connection within their fields, perhaps even helping to reduce imposter syndrome; and yet, such feelings of belonging might often be difficult to achieve, as demonstrated in Study 1. Certainly, to the degree that women feel identified with their discipline, they also tend to show better mental health, but the question remains how such identification can be fostered in the first place. It may be that change must occur within some (especially male-dominated) disciplines to ensure that maintaining a strong sense of disciplinary identity is possible among often out-numbered postdoctoral women. Likewise, it is worth noting that these findings may also have implications for scholars who engage in (increasingly common and encouraged) interdisciplinary work (Nissani, 1997; Bammer, 2017), including (and especially) the field of health research itself (Jacobs and Frickel, 2009), with strategies to ensure that those women’s primary disciplinary identities are not lost.

Despite the often-acknowledged importance of gender identification—including within academia and male-dominated fields (Kaiser and Spalding, 2015; Hernandez et al., 2017), only 5.6% of women rated gender as their most important social identity in our online study (with social, work, and parental identities far exceeding this number). Moreover, gender identification was unrelated to women’s perceived control and mental health in Study 2. This may reflect traditional self-categorization processes (Turner et al., 1994), such that women’s disciplinary identities were relatively more salient while completing a survey about health and work, and thus was a stronger predictor in this context. It may be that the other identities we assessed—including gender and social identification—would prove beneficial in other non-work contexts, or when gender itself is made particularly salient (e.g., in circumstances involving sexism, tokenism, or intersectional identities). Indeed, given the comparative nature of simultaneously assessing numerous identities at once, gender identity may simply have been considered less relevant here. However, given an institutional climate in which women are often under-represented, especially at more senior levels (The Lancet, 2018), equally plausible is that women’s gender identity was indeed quite salient but felt targeted, and likewise failed to predict well-being.

It is also important to consider the notion of multiple group memberships and identities, which have been shown in many instances to be protective of mental health (e.g., Ysseldyk et al., 2013; Jetten et al., 2015; Miller et al., 2017). The message here would be that women should not feel obligated to choose their disciplinary or gender (or any other) identity at the expense of another important group membership or life role (e.g., identifying as a parent). Unfortunately, however, as noted by the women in Study 1, the academic culture often leaves little room for such work-life flexibility. Given previous research on the potential for incompatibility (Iyer and Ryan, 2009; Cruwys et al., 2016; Matschke and Fehr, 2017; Sønderlund et al., 2017), interference (Settles, 2004), and complex intersectionality (Roccas and Brewer, 2002; Collins, 2015) among identities, new strategies—at individual, group, and institutional levels—may be needed to ensure that women’s multiple group memberships and identities (and associated well-being) can be maintained within academia.

### Implications for Workplace Equality Within the Academy

Much previous research has focussed on addressing gender inequality (for a recent overview, see Morgenroth and Ryan, 2018), including within academia (e.g., Kinman, 2001; Savigny, 2014; Lee, 2015; Howe-Walsh and Turnbull, 2016; Boring, 2017; Howes et al., 2018; Lundine et al., 2018). And yet, the struggles of postdoctoral women specifically—women who are arguably at the greatest risk for opting out of an academic career despite a decade (or more) of working toward it—have often been overlooked (cf. Goulden et al., 2011; Case and Richley, 2013; Ledford, 2017). The present research thus fills an important gap by identifying and addressing the issue of the leaky pipeline where it may be the most susceptible and, importantly, by collecting evidence from the target’s perspective—the perspectives of postdoctoral women themselves.

Not with standing the potential pitfalls of putting all of one’s proverbial group identification eggs into one basket, the notion of fostering strong disciplinary identities suggests a potential intervention strategy for helping to keep postdoctoral women from slipping through the leaky pipeline. While it is beyond the scope of this research, the social meaning of disciplinary identity varies across academia, with implications for gender inequality; for example, in mathematics and physics, the expectation that scholars possess innate “brilliance” and make their key contributions early in their careers exacerbates gender inequities in these fields (Leslie et al., 2015). Our data suggest that in addition to improving women’s mental health outcomes, a strong sense of belonging and inclusion may also cultivate a sense of control over the career uncertainty that so often plagues postdoctoral women. Importantly, however, this should not be on the shoulders of postdocs alone; instead, institutional culture change—from the individual to the ivory tower—must occur in order to promote a sense of inclusion and respect for (and among) women. As noted by the women we interviewed, this includes respect for women’s research and ideas in male-dominated environments (Young et al., 2013) as well as putting competition secondary to advancing reciprocal support among women themselves. In short, it is not a matter of “fixing” women to feel more comfortable with job insecurity, but fixing the academic system to better support and protect mental health among vulnerable participants.

Most unique to the postdoctoral stage, however, may be institutional policies (or lack thereof; Horton, 2018) related to maternity leave specifically. Although gender inequality within academia affects women with and without children in many respects (e.g., teaching ratings, publication bias, tenure and promotion rates), the “baby penalty” inherent in taking maternity leave at the postdoctoral stage has been noted as especially problematic in previous research (Mason and Goulden, 2004; Ledford, 2017). Indeed, this was also expressed repeatedly by the women we interviewed, and intersected with the themes of sexism, work-life balance, and uncertainty. Interestingly, however, fears associated with the stigma of taking maternity leave, as well as attempting to balance being a parent and an academic, were voiced by both parents and non-parents alike. In this regard, women without children conveyed worry over whether or when they should plan to have children, or relief over still being “safe” because they did not yet have them. Moreover, the problem of losing postdoctoral mothers to the leaky pipeline was also reflected in the glaringly low number of mothers who participated in our research—37% in the online survey, and only 28% of the women interviewed. These figures, along with the concerns expressed by the women in our research, lend further evidence that many mothers may opt out of an academic career before it even begins, conceivably due to inequitable institutional policies or the inevitable penalty in academic output that lead them to conclude that an academic career is not amenable to family life.

### Caveats and Limitations

Like all research, the studies we present here have limitations. First, responses to our online survey were only received from Australia, Europe, and North America, and the total sample size of women currently in a postdoctoral position was somewhat small; while this sample size was adequate for our analyses and does represent a broad array of nationalities, we should not generalize our results to postdoctoral women from Asian or African institutions, or elsewhere. Likewise, although we aimed to collect data from a variety of early career researchers, including men and tenure-track professors, perhaps due to our recruitment strategies (or due to the impetus of certain demographics to complete our survey), the bulk of our sample constituted postdoctoral women, thus making comparative analyses (e.g., with postdoctoral men) untenable. And finally, as with all cross-sectional survey data, causal conclusions should be interpreted cautiously (e.g., the links between group identification and mental health may be reciprocal; Miller et al., 2017).

Similarly, our interviews were restricted to Canada and Germany, and thus might not fully represent the views of postdoctoral women in other countries. Nonetheless, despite being an ocean apart, many of the challenges faced by women in these two countries were strikingly similar. The women interviewed were also primarily Caucasian, and so some issues related to intersectionality (Collins, 2015) and the “concrete ceiling” (Cotter et al., 2001) for women from racial or ethnic minority groups could not be fully explored. Nonetheless, in both our online survey and interviews, we were able to gain the perspectives of postdoctoral women from a broad range of disciplinary backgrounds and experiences. Indeed, the mixed-method approach was also a strength of our research. Several of the themes from the qualitative insights drawn from our interviews were affirmed in our quantitative analyses, including the strained mental health of postdoctoral women, the importance of feeling a sense of support and belonging—especially among one’s academic peers—and the relative *lack* of reinforcement gained by identifying with one’s gender group alone.

## Conclusion

Women face barriers to achieving equitable representation in many professions, and academia is no exception. Within this often-stressful environment, the postdoctoral years pose a specific challenge brought about by a cocktail of job insecurity, identity uncertainty, and concurrent life changes. Available data suggest that women are at risk of falling prey to the leaky academic pipeline at the postdoctoral stage. Our qualitative study outlined various barriers faced by postdoctoral women “in their own words,” including implications for mental health. A follow-up study quantified these mental health issues in a larger sample of postdoctoral women, highlighting disciplinary identification as a protective factor in the academic environment. Together, the results suggest that a sense of belonging is critical for combating the forces that contribute to a decision to exit academic life. But as with many gender-based investigations, the main implication of our findings is not that women should be made to change or adapt to less than ideal circumstances. Rather, we argue that structural sexism within the system itself should be adapted in order to remove the barriers that contribute to academic workplace inequality, particularly at (but not limited to) the postdoctoral stage where the pipeline may leak the most.

## Ethics Statement

This research was carried out in accordance with the recommendations of the Tri-Council Policy Statement on the Ethical Conduct for Research Involving Humans by the Carleton University Research Ethics Board-B (CUREB-B) with written informed consent from all participants. The protocol was approved by the CUREB-B.

## Author Contributions

RY, KG, EH, MB, MF, ES, and VT developed the study concept and designed the research. RY performed the qualitative analyses for Study 1 and drafted the manuscript. KG performed the statistical analyses for Study 2. SZ conducted and transcribed the Canadian interviews, and managed the online data collection. JL conducted and transcribed the German interviews. All authors edited, read, and approved the final manuscript.

## Conflict of Interest Statement

The authors declare that the research was conducted in the absence of any commercial or financial relationships that could be construed as a potential conflict of interest.

## Footnotes

1 Although all interviews were conducted in Canada and Germany, these postdoctoral women were citizens of a variety of countries. Nonetheless, for ease of presentation and to protect anonymity, participants are referred to as “Canadian postdoc” or “German postdoc” throughout this article.

2 The themes that emerged from the samples in Canada and Germany overlapped to the extent that composite results for both samples are presented here, with any nuances noted.
